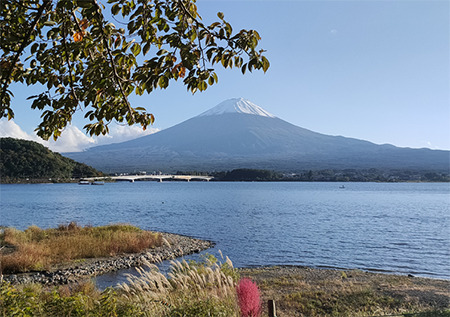# Never lose hope, regardless of how gloomy it gets – Fuji-san

**DOI:** 10.51866/mol.981

**Published:** 2025-09-11

**Authors:** Imran Ahmad, Ruzilawati Abu Bakar

**Affiliations:** 1 BMedSc, MBBS, MMed (Fam Med), Department of Family Medicine, School of Medical Sciences, Universiti Sains Malaysia, Kubang Kerian, Kelantan, Malaysia. Email: profimran@usm.my; 2 PhD, Department of Pharmacology, School of Medical Sciences, Universiti Sains Malaysia, Kubang Kerian, Kelantan, Malaysia.

**Keywords:** Hopelessness, Luck, Primary care physician

My wife and I attended the Human Genetics Asia 2023 Congress held in Tokyo, Japan, in October 2023. We were accompanied by our two daughters. For the three, it was their first visit to Tokyo. For me, it was my third. The congress was well-attended by both local and international delegates. We found it fulfilling to listen to the lectures and discussions. More importantly, we valued the opportunity to meet and converse with the attending delegates. We also managed to present our two papers at the congress.

Naturally, our visit was not all about science. While in Tokyo, we took the chance to savour the city’s beauty. I had a specific mission this time: to finally capture a perfect selfie with the world’s shyest mountain, Fuji-san or Mount Fuji. My first attempt, back in 2015, failed because the peak was hidden behind a ghostly cloud. The second one was even worse: Our tour bus did not go anywhere near Mount Fuji because of heightened seismic activity in the region. This third time appeared equally unpromising for different reasons.

We resigned ourselves to not having the best view of Mount Fuji that day. When we left our guesthouse in Oshiage that morning, it was drizzling, and the sky was overcast. Our mood matched the gloomy weather; on a day like this, there is typically little chance Mount Fuji would make an appearance. Nonetheless, we walked to the nearby station and boarded the train. We stopped at Shibuya Station and made our way to the bus terminal. Then came another disappointment: We missed the only morning bus from Shibuya to Kawaguchiko. My chances of seeing Mount Fuji on this third attempt seemed slimmer than before.

With little hope left, we boarded the train again, this time trying our luck in Shinjuku. Since Shinjuku has more bus departures, we managed to catch one and were finally on our way to Kawaguchiko. The drizzle had stopped by the time we arrived at Kawaguchiko Station, but the sky remained heavy with grey clouds. We boarded the sightseeing bus and were content with the beautiful scenery along the way. The three girls were happy to catch a glimpse of Mount Fuji, albeit with its peak hidden behind the clouds. To them, it was still Mount Fuji. As for me, I was already daydreaming about my fourth attempt to see it in full.

Suddenly, after passing a few stations, we saw Mount Fuji in its full glory through the window of the moving bus. We alighted the bus at the next stop and rushed to find the best viewing spot. As the saying goes, the rest was history. We took countless photos with Mount Fuji. More importantly, we learnt the best lesson that day, one different from what we gained at the conference. This lesson was deeper: Never give up. Mount Fuji taught us that. This message is also for all young and aspiring primary care physicians. Never lose hope, regardless of how gloomy it gets.

**Figure:**
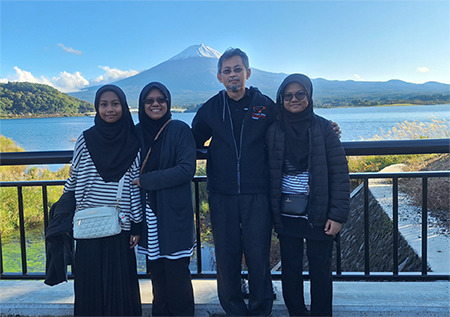


**Figure:**